# Hepatitis C Virus Clearance and Diffusing Capacity for Carbon Monoxide in Women With and Without Human Immunodeficiency Virus

**DOI:** 10.1093/ofid/ofae251

**Published:** 2024-05-02

**Authors:** Andrew C Curnow, Laurence Huang, Margaret A Fischl, Michelle Floris-Moore, Alison Morris, Mehdi Nouraie, Divya B Reddy, Eric C Seaberg, Anandi N Sheth, Phyllis C Tien, Richard J Wang

**Affiliations:** Department of Medicine, University of California, San Francisco, San Francisco, California, USA; Department of Medicine, San Francisco Veterans Affairs Health Care System, San Francisco, California, USA; Department of Medicine, University of California, San Francisco, San Francisco, California, USA; Department of Medicine, University of Miami Miller School of Medicine, Miami, Florida, USA; Department of Medicine, University of North Carolina School of Medicine, Chapel Hill, North Carolina, USA; Department of Medicine, University of Pittsburgh School of Medicine, Pittsburgh, Pennsylvania, USA; Department of Medicine, University of Pittsburgh School of Medicine, Pittsburgh, Pennsylvania, USA; Department of Medicine, Albert Einstein College of Medicine, Bronx, New York, USA; Department of Epidemiology, Johns Hopkins University, Baltimore, Maryland, USA; Department of Medicine, Emory University School of Medicine, Atlanta, Georgia, USA; Department of Medicine, University of California, San Francisco, San Francisco, California, USA; Department of Medicine, San Francisco Veterans Affairs Health Care System, San Francisco, California, USA; Department of Medicine, University of California, San Francisco, San Francisco, California, USA

**Keywords:** antiviral agents, hepatitis C, human immunodeficiency virus, pulmonary diffusing capacity

## Abstract

Hepatitis C virus (HCV) infection is associated with extrahepatic effects, including reduced diffusing capacity of the lungs. It is unknown whether clearance of HCV infection is associated with improved diffusing capacity. In this sample of women with and without human immunodeficiency virus, there was no association between HCV clearance and diffusing capacity.

Hepatitis C virus (HCV) infection is associated with extrahepatic injury, including reduced diffusing capacity for carbon monoxide (DLCO) [1, 2], which is a measure of respiratory gas exchange that is decreased in parenchymal lung diseases, such as emphysema, interstitial lung diseases, and pulmonary hypertension. Among persons living with human immunodeficiency virus (HIV), which itself is associated with reduced DLCO, HCV seropositivity is also independently associated with diffusion impairment [2–5]. While HIV viral suppression after antiretroviral treatment is associated with higher DLCO, the effects of HCV treatment on DLCO are unknown [4]. One study suggested that untreated, chronic HCV infection was associated with a more rapid decline in DLCO among people with chronic obstructive pulmonary disease [6]. In this study, we examined the relationship between treatment-related and spontaneous HCV clearance with DLCO in a cohort of women with and without HIV. We hypothesized that clearance of HCV infection, either spontaneously or with treatment, would be associated with higher DLCO measurements compared to ongoing HCV viremia.

## METHODS

This was a cross-sectional analysis of participants in the Women's Interagency HIV Study (WIHS) who had single-breath DLCO measurements collected between 2018 and 2019 as part of a pulmonary function ancillary study. The WIHS is a cohort study of women with and at risk for HIV infection who were enrolled in 4 waves, the first in 1994 and the last in 2013. Details regarding the WIHS cohort have been detailed elsewhere [7–9]. Participants were excluded from the analysis if they were pregnant, did not have diffusing capacity measurements that met standards for quality and reproducibility, or reported undergoing HCV treatment at the time of DLCO measurement.

At the time of enrollment into the WIHS, participants underwent anti-HCV serologic testing, and if participants tested HCV seropositive, serum HCV RNA testing was performed. At each semi-annual WIHS clinical research visit, HCV-seropositive participants were asked about HCV treatment. For participants who reported receiving HCV treatment, serum HCV RNA was measured 6 months or more after treatment completion.

HCV-seropositive participants were classified as undetectable HCV RNA without HCV treatment (spontaneously cleared), undetectable HCV RNA after HCV treatment (successfully treated), or detectable HCV RNA regardless of HCV treatment (viremic).

The primary outcome measurement was DLCO. Percent-of-predicted DLCO and DLCO *z* scores were calculated based on standard reference equations and analyzed as secondary outcomes [10–12].

Five main comparisons were planned. First, HCV-seropositive participants were compared to seronegative participants. Second, among HCV-seropositive participants, HCV-viremic participants were compared to nonviremic participants (spontaneously cleared or successfully treated). Third, among nonviremic participants, participants who were successfully treated for HCV infection were compared to participants with spontaneously cleared HCV infection. Fourth, among HCV-treated participants, we compared participants who were or were not exposed to either interferon alfa, ribavirin, or both. Last, HCV-viremic participants were compared to participants who were successfully treated for HCV infection. The *t* test was used for univariate analysis. Multivariable linear regression modeling adjusted for the following: age, height, hemoglobin and carboxyhemoglobin concentration, education level, HIV infection, self-reported cigarette smoking (pack-years and current smoking status), and self-reported heroin, cocaine, and cannabis use [13–16].

## RESULTS

Among the 669 eligible participants included in the analysis, 133 were HCV seropositive (97 with HIV coinfection), 365 had HIV monoinfection, and 171 had neither infection. Twenty-four of the 133 HCV-seropositive participants were viremic, while 48 were categorized as spontaneously cleared HCV and 61 as successfully treated for HCV (15 with exposure to interferon alfa and/or ribavirin) (Table 1).

**Table 1.** Baseline Characteristics for Participants With Diffusing Capacity Measurements

Characteristic	HCV Seronegative (n = 536)	HCV Seropositive (n = 133)		
		Viremic (n = 24)	Not Viremic	
			No Treatment (n = 48)	Exposed to Treatment (n = 61)
Age, y, median (IQR)	49 (42–55)	54 (50–57)	58 (53–61)^a,b^	59 (55–62)^a,b^
Height, cm, median (IQR)	163 (158–167)	160 (158–167)	161 (157–165)	163 (158–165)
Hemoglobin, g/dL, median (IQR)	12.7 (11.9–13.6)	12.8 (11.9–13.5)	13.1 (11.9–13.8)	13.1 (12.4–13.8)^a^
Carboxyhemoglobin, %, median (IQR)	3 (2–5)	5.5 (4–7)^a^	4 (2.5–6)	5 (3–6)^a^
Race			^a^	
Black	368 (69)	14 (58)	21 (44)	38 (62)
White	52 (10)	2 (8)	15 (31)	8 (13)
Other	116 (22)	8 (33)	12 (25)	15 (25)
Education				
Did not complete high school	167 (31)	10 (42)	12 (25)	26 (43)
Smoking		^a^	^a,b^	^a,b^
Never	182 (34)	2 (8)	9 (19)	5 (8)
Former	216 (40)	19 (79)	20 (42)	32 (52)
Current	138 (26)	3 (13)	19 (40)	24 (39)
Smoking pack-years^c^, median (IQR)	8.1 (3.2–14.6)	12.2 (6.3–17.6)	18.9 (9.3–27.1)^a,b^	13.3 (8.6–19.7)
Marijuana use				
Current	164 (31)	12 (50)	17 (35)	17 (28)
Ever	378 (71)	21 (88)	43 (90)^a^	54 (89)^a^
Cocaine use				
Current	43 (8)	6 (25)^a,b^	4 (8)	3 (5)
Ever	280 (52)	19 (80)^a^	35 (73)^a^	49 (80)^a^
Heroin or other IDU				
Current	3 (1)	3 (13)^a^	3 (6)^a^	1 (2)
Ever	55 (10)	13 (54)^a^	33 (69)^a^	44 (72)^a^
HIV				
Living with HIV	365 (68)	11 (46)^a^	38 (79)^b^	48 (79)^b^
HIV RNA undetectable^d^	225 (62)	4 (45)	28 (68)	31 (65)
Currently on ART^d^	336 (92)	9 (82)	36 (95)	45 (94)
Nadir CD4 count^d^, cells/μL, median (IQR)	237 (88–394)	199 (125–390)	212 (97–328)	204 (108–310)
History of *Pneumocystis* pneumonia^d^	16 (4)	0 (0)	3 (8)	5 (10)
History of non-*Pneumocystis* pneumonia^d^	48 (13)	3 (27)	10 (26)^a^	18 (38)^a^
HCV treatments				^b^
Ribavirin and/or interferon alfa only	…	2 (8)	…	9 (15)
DAAs only	…	1 (4)	…	46 (75)
Both DAAs and ribavirin and/or interferon alfa	…	0 (0)	…	6 (10)
Hepatitis B infection	12 (2)	0 (0)	3 (6)	1 (2)
APRI score, median (IQR)	0.18 (0.13–0.24)	0.31 (0.22–0.59)^a^	0.24 (0.16–0.35)^a^	0.24 (0.17–0.30)^a^
FIB-4 score, median (IQR)	0.86 (0.63–1.1)	1.3 (0.85–2.2)^a^	1.3 (0.95–1.8)^a^	1.3 (1.0–1.9)^a^
FibroScan liver elastography, kPa, median (IQR) (n = 489)	5.0 (3.8–6.1)	6.8 (6.0–8.9)^a^	5.2 (4.3–7.0)^a^	6.4 (4.5–8.5)^a^

Data are presented as No. (%) unless otherwise indicated.

Abbreviations: ART, antiretroviral therapy; APRI, aspartate aminotransferase to platelet ratio index; DAA, direct-acting antiviral; FIB-4, Fibrosis-4 Index; HCV, hepatitis C virus; HIV, human immunodeficiency virus; IDU, intravenous drug use; IQR, interquartile range.

^a^Compared to HCV-seronegative group, *P* value for *t* test or Fisher exact test <.05.

^b^Compared to HCV-viremic group, *P* value for *t* test or Fisher exact test <.05.

^c^Current and former smokers only.

^d^Among persons with HIV only.

Compared to HCV-seronegative participants, seropositive participants had lower DLCO (adjusted mean difference, −1.2 mL/minute/mm Hg [95% confidence interval {CI}, −1.9 to −.4]), lower percent-of-predicted DLCO (adjusted mean difference of −5% of predicted [95% CI, −9% to −1%]), and lower DLCO *z* score (adjusted mean difference, −0.71 [95% CI, −1.05 to −.37]) (Table 2). Participants with HCV viremia had a higher DLCO than those without viremia (unadjusted mean difference, +1.9 mL/minute/mm Hg [95% CI, +.2 to +3.7) and a higher percent-of-predicted DLCO (unadjusted mean difference, +9% of predicted [95% CI, +1 to +17]), but the adjusted differences were not statistically significant (adjusted mean difference, +1.1 mL/minute/mm Hg [95% CI, −.6 to +2.7] and +6% of predicted [95% CI, −2 to +15]); there was no significant differences in DLCO *z* scores. We did not observe significant differences between participants with successfully treated and spontaneously cleared HCV infection, or between participants who were or were not exposed to interferon alfa and/or ribavirin (Table 2).

**Table 2.** Differences in Diffusing Capacity Between Exposed and Unexposed Groups

Comparison	Exposed Group	DLCO (mL/min/mm Hg), Mean (SD)	Unadjusted Mean Difference (95% CI)	Adjusted Mean Difference^a^ (95% CI)	Percent Predicted DLCO, Mean (SD)	Unadjusted Mean Difference (95% CI)	Adjusted Mean Difference^b^ (95% CI)	DLCO *z* Score, Mean (SD)	Unadjusted Mean Difference (95% CI)	Adjusted Mean Difference^b^ (95% CI)
	Comparison Group									
HCV seropositivity	Seropositive (n = 133)	15.1 (4.0)	**−2.7 (−3.4 to −1.9)**	**−1.2 (−1.9 to −.4)**	78 (18)	**−10 (−13 to −6)**	**−5 (−9 to −1)**	−2.07 (1.70)	**−0.78 (−1.07 to −.49)**	**−0.71 (−1.05 to −.37)**
	Seronegative (n = 536)	17.7 (4.1)	***P* < .0001**	***P* = .004**	88 (17)	***P* < .0001**	***P* = .01**	−1.29 (1.47)	***P* < .001**	***P* < .001**
HCV viremia	Viremic (n = 24)	16.6 (4.5)	**1.9 (.2–3.7)**	1.1 (−.6 to 2.7)	86 (18)	**9 (1–17)**	6 (−2 to 15)	−1.51 (1.37)	0.68 (−.07 to 1.43)	0.77 (−.06 to 1.59)
	Not viremic (n = 109)	14.7 (3.8)	***P* = .03**	*P* = .20	77 (18)	***P* = .03**	*P* = .16	−2.19 (1.74)	*P* = .07	*P* = .07
HCV treatment status	Prior HCV treatment history (n = 61)	14.5 (3.5)	−0.5 (−2.0 to .9)	0.3 (−1.1 to 1.7)	75 (17)	−3 (−10 to 4)	1 (−6 to 8)	−2.26 (1.65)	−0.16 (−.83 to .51)	0.21 (−.53 to .96)
	No prior HCV treatment history (n = 48)	15.0 (4.1)	*P* = .46	*P* = .41	78 (20)	*P* = .39	*P* = .81	−2.10 (1.87)	*P* = .63	*P* = .58
Exposure to interferon and/or ribavirin	Exposed to interferon/ribavirin (n = 15)	15.7 (4.2)	1.6 (−.5 to 3.7)	1.8 (−.4 to 4.0)	82 (19)	9 (−1 to 18)	11 (−1 to 22)	−1.75 (2.04)	0.67 (−.30 to 1.65)	0.69 (−.43 to 1.82)
	Not exposed to interferon/ribavirin (n = 46)	14.1 (3.2)	*P* = .12	*P* = .11	73 (16)	*P* = .09	*P* = .07	−2.43 (1.49)	*P* = .17	*P* = .22

Estimates for which the *P* value is less than 0.05 are in bold. Abbreviations: CI, confidence interval; DLCO, diffusing capacity for carbon monoxide; HCV, hepatitis C virus; SD, standard deviation.

^a^Adjusted for age, height, education level, hemoglobin, carboxyhemoglobin, human immunodeficiency virus (HIV) infection, self-reported cigarette smoking (measured in pack-years and current smoking status), and self-reported heroin, cocaine, and cannabis use.

^b^Adjusted for age, education level, HIV infection, self-reported cigarette smoking (measured in pack-years and current smoking status), and self-reported heroin, cocaine, and cannabis use.

When comparing participants with HCV viremia to participants who were successfully treated, there was no significant difference in DLCO (adjusted mean difference, +1.0 mL/minute/mm Hg [95% CI, −.94 to +3.0]), percent-of-predicted DLCO (adjusted mean difference, +5% of predicted [95% CI, −5 to +15]), or DLCO *z* score (adjusted mean difference, +0.76 [95% CI, −.12 to +1.63]).

Results were not meaningfully different when analyses were restricted only to participants living with HIV.

## DISCUSSION

In this multisite, racially, and ethnically diverse cohort of United States women with and without HIV infection, we found a robust association between hepatitis C seropositivity and diffusion impairment, consistent with prior studies [2, 3]. Contrary to our hypothesis, we did not observe that women with treated or spontaneously cleared HCV infection had a higher DLCO compared to women with HCV viremia.

By contrast, the point estimate for this analysis indicates that participants with HCV viremia had a higher mean DLCO than participants without HCV viremia after adjustment for confounding exposures, although the relationship was not statistically significant. We think it is biologically implausible that HCV viremia has a salutary effect on diffusing capacity, so we entertain 2 possibilities.

The first possibility is that HCV viral clearance truly has no effect on DLCO. While we and others show that HCV seropositivity is associated with lower DLCO, our analysis by HCV viremia status could indicate that acute HCV infection has a long-lasting effect on DLCO despite spontaneous or treatment-related HCV clearance. This kind of durable, extrahepatic consequence has been observed with HCV-associated chronic kidney disease and HCV-associated cryoglobulinemia, which persist even in the absence of detectable HCV RNA [17–21].

The second possibility is that HCV viral clearance can improve DLCO as hypothesized, akin to its reported effect on diabetes and cardiac disease risk, but we did not observe this because of chance, modeling error, unmeasured confounding, or some combination thereof [22–25]. The overall prevalence of untreated HCV infection in the United States has decreased due to the availability of direct-acting antiviral treatment, and the lack of exchangeability between viremic and nonviremic groups may persist even after conditioning our analyses on possible confounding variables [26]. In particular, the proportion of participants with HCV viremia who also had HIV coinfection is lower than in the comparator groups, and there is robust evidence that HIV infection is a cause of diffusion impairment [3–5]; although HIV infection was accounted for in multivariable regression modeling, the possibility of incomplete adjustment and residual confounding persists. Furthermore, data on the duration of HCV infection are unavailable in this cohort, and it is possible that participants found to have HCV viremia were, on average, more recently infected—and, therefore, exposed to HCV viremia for a shorter lifetime duration—than participants who had cleared infection due to successful treatment. As such, modeling HCV viremia as a binary variable may explain the paradoxical finding of its unadjusted association with a higher DLCO.

This analysis is the first to examine the relationship between HCV viral clearance and diffusing capacity. While we do not report any definitive conclusion, we believe that our findings raise an important question with implications for the long-term respiratory health of persons with cleared HCV infection and merits further investigation. Strengths of this study include a well-characterized sample population of women with a high HCV seroprevalence. Limitations include the cross-sectional study design, the unavailability of data on the duration of HCV infection, and the small number of women with HCV viremia.

## Notes

***Author contributions.*** Conception and design: A. C. C., P. C. T., and R. J. W. Data analysis: A. C. C., P. C. T., and R. J. W. Interpretation: A. C. C., L. H., M. A. F., M. F.-M., A. M., M. N., D. B. R., E. C. S., A. N. S., P. C. T., and R. J. W. Manuscript draft: A. C. C. and R. J. W. Manuscript revision and approval of manuscript: All authors.

***Acknowledgments.*** The authors gratefully acknowledge the contributions of the study participants and dedication of the staff at the Multicenter AIDS Cohort Study/Women’s Interagency HIV Study (WIHS) Combined Cohort Study (MWCCS) sites.

***Patient consent.*** The WIHS protocol was approved by the institutional review board at each participating site (Albert Einstein College of Medicine, University of California, San Francisco, University of North Carolina at Chapel Hill, Emory University, and University of Miami). All participants provided written informed consent.

***Financial support*.** Data in this manuscript were collected by the WIHS, now the MWCCS, which is supported by the National Institutes of Health. Full acknowledgement may be found here: https://statepi.jhsph.edu/mwccs/acknowledgements/. The study was also supported by the National Heart, Lung, and Blood Institute (award numbers K12 HL143961 and K23 HL162593 to R. J. W.) and the National Institute of Allergy and Infectious Diseases (award number K24 AI108516 to P. C. T.; grant number P30 AI094189 to E. C. S.).
